# Electrophysiological evidence for functionally distinct neuronal populations in the human substantia nigra

**DOI:** 10.3389/fnhum.2014.00655

**Published:** 2014-09-09

**Authors:** Ashwin G. Ramayya, Kareem A. Zaghloul, Christoph T. Weidemann, Gordon H. Baltuch, Michael J. Kahana

**Affiliations:** ^1^Department of Neuroscience, Neuroscience Graduate Group, University of PennsylvaniaPhiladelphia, PA, USA; ^2^Surgical Neurology Branch, National Institutes of Neurological Disorders and Stroke, National Institutes of HealthBethesda, MD, USA; ^3^Department of Psychology, Swansea UniversitySwansea, UK; ^4^Department of Neurosurgery, Perelman School of Medicine, University of PennsylvaniaPhiladelphia, PA, USA; ^5^Department of Psychology, University of PennsylvaniaPhiladelphia, PA, USA

**Keywords:** substantia nigra, human, dopamine, GABA, neuron, reinforcement learning

## Abstract

The human substantia nigra (SN) is thought to consist of two functionally distinct neuronal populations—dopaminergic (DA) neurons in the *pars compacta* subregion and GABA-ergic neurons in the *pars reticulata* subregion. However, a functional dissociation between these neuronal populations has not previously been demonstrated in the awake human. Here we obtained microelectrode recordings from the SN of patients undergoing deep brain stimulation (DBS) surgery for Parkinson's disease as they performed a two-alternative reinforcement learning task. Following positive feedback presentation, we found that putative DA and GABA neurons demonstrated distinct temporal dynamics. DA neurons demonstrated phasic increases in activity (250–500 ms post-feedback) whereas putative GABA neurons demonstrated more delayed and sustained increases in activity (500–1000 ms post-feedback). These results provide the first electrophysiological evidence for a functional dissociation between DA and GABA neurons in the human SN. We discuss possible functions for these neuronal responses based on previous findings in human and animal studies.

## 1. Introduction

Animal studies have shown that the substantia nigra (SN) consists of two functionally distinct neuronal populations—dopaminergic (DA) neurons in the *pars compacta* subregion and GABA-ergic neurons in the *pars reticulata* subregion. DA neurons have been shown to encode reward prediction errors with phasic bursts of firing, that occur when there is a mismatch between obtained and expected outcomes (Schultz et al., 1997; Bayer and Glimcher, 2005). These DA bursts are thought to guide reinforcement learning by adjusting synaptic strength in downstream regions following unexpected outcomes (Reynolds et al., 2001; Tsai et al., 2009). In contrast, GABA neurons are involved in inhibitory regulation of various brain structures including frontal cortical regions (via the thalamus), premotor brainstem nuclei and midbrain DA neurons (Carpenter et al., 1976; Hikosaka and Wurtz, 1983; Tepper et al., 1995; Henny et al., 2012). Despite these advances in the animal, the functional role of human SN neurons has not been elucidated.

Patients undergoing deep brain stimulation (DBS) surgery for the treatment of Parkinson's Disease offer a rare opportunity to directly study the functional properties of human SN neurons (Jaggi et al., 2004). Two previous studies in patients undergoing DBS suggest a functional role for the human SN in reinforcement learning. First, it has been shown that a subset of neurons in the SN demonstrate phasic bursts of activity following unexpected rewards, consistent with a reward prediction error (Zaghloul et al., 2009). Second, microstimulation applied in the SN following rewards alters learning by enhancing the reinforcement of preceding actions (Ramayya et al., 2014). In both studies, the observed learning-related neural and behavioral patterns were presumed to reflect the function of a healthy subpopulation of DA neurons in the region. Although histochemical studies have shown that DA and GABA neurons co-exist in the human SN (Damier et al., 1999a), a functional dissociation between these SN neural populations has not previously been demonstrated.

In this study, we sought to directly compare the response profiles of DA and GABA neurons recorded from the human SN so as to assess whether these neuron groups represent functionally distinct subpopulations. We obtained recordings from 25 subjects as they performed a two-alternative reinforcement learning task where they selected between stimuli that carried distinct reward probabilities and received positive or negative feedback following each choice. We extracted neuronal spiking activity from each unit and identified putative DA and GABA neurons based on the physiological properties of their recorded waveforms (Joshua et al., 2009; Matsumoto and Hikosaka, 2009; Ungless and Grace, 2012). If DA and GABA neurons demonstrate distinct task-related responses, it would suggest that they represent functionally distinct neuronal populations.

## 2. Materials and methods

### 2.1. Electrophysiological recordings

**Figure 1:**
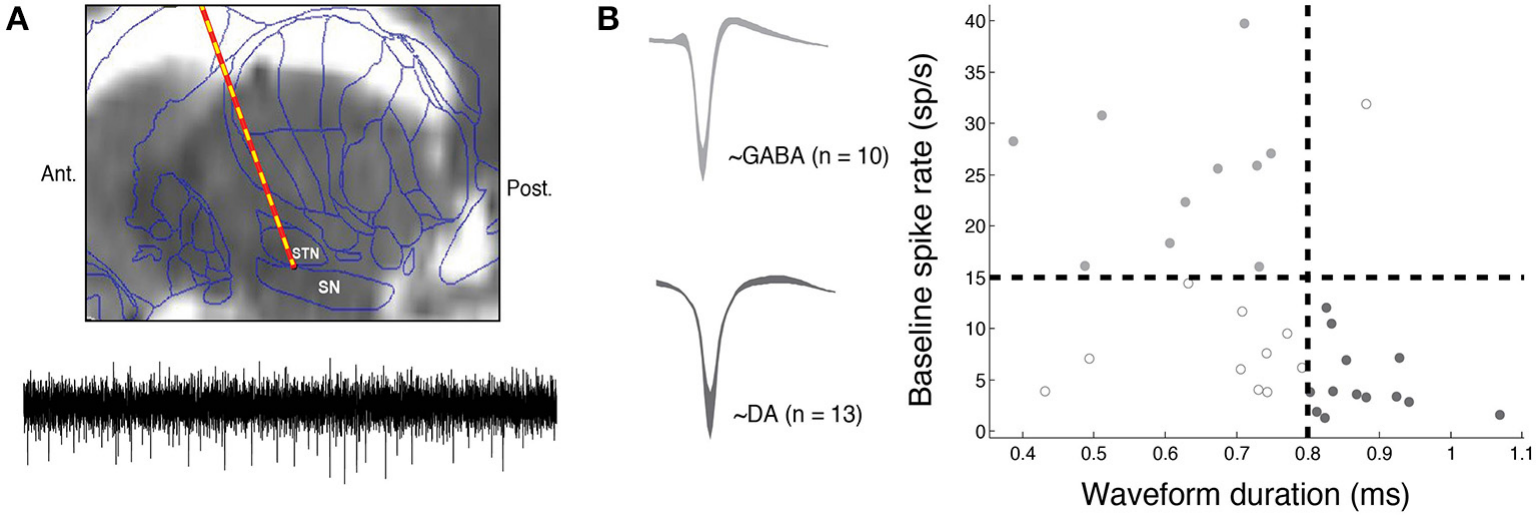
**Intra-operative electrophysiological methods. (A)** During deep brain stimulation (DBS) surgery, a microelectrode is advanced into the substantia nigra (SN) to identify the inferior border of the subthalamic nucleus (STN). Top–an example pre-operative MRI scan (sagittal view) overlaid with a standard brain atlas and estimated microelectrode position is shown. This figure is adapted from Zaghloul et al. (2009). Bottom–an example 500 ms band-pass (400–3000 Hz) filtered voltage trace is shown. We extracted neuronal spiking activity from each microelectrode recording by identifying spikes in the filtered signal that demonstrated sufficient separation from background noise (Materials and Methods). **(B)** We identified putative DA (*n* = 13, dark gray) and GABA (*n* = 10, light gray) units based on their baseline firing rate and waveform durations. Left: mean waveforms from DA and GABA fast-spiking units. Width represents standard error of mean (s.e.m). Units that did not fall in either category are marked with open circles.

We obtained intra-operative microelectrode recordings from 25 Parkinsonian patients undergoing surgery for the implantation of a deep brain stimulator (DBS) in the subthalamic nucleus (STN). Patients who volunteered to take part in the study provided their informed consent during preoperative consultation and received no financial compensation for their participation. Per routine clinical protocol, Parkinson's medications were stopped on the night before surgery (12 h preoperatively); hence subjects engaged in the study while in an OFF state. The study was conducted in accordance with a University of Pennsylvania Institutional Review Board-approved protocol. During surgery, intra-operative microelectrode recordings (obtained from a 1 μm diameter tungsten tip electrode advanced with a power-assisted microdrive) were used to identify the substantia nigra (SN) and the STN as per routine clinical protocol. We obtained microelectrode recordings sampled at 25 kHz using a StimPilot recording system (16 bit analog-to-digital converter) and Spike2 data acquisition software (Figure 1; targeting and recording details are reported elsewhere; Moyer et al., 2007). In this study, we present data captured from the SN as subjects performed the reinforcement learning task described below (see “Reinforcement learning task”).

### 2.2. Reinforcement learning task

**Figure 2:**
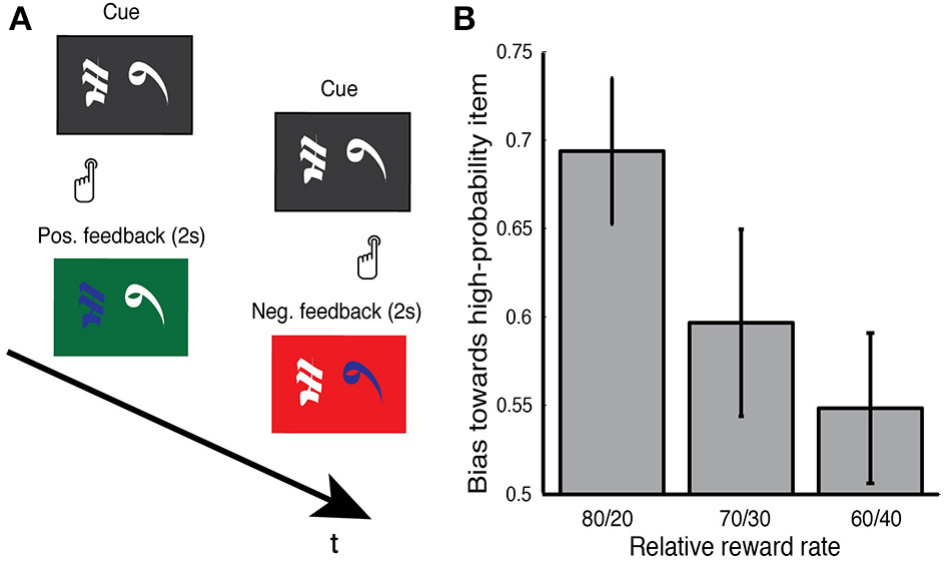
**(A)** Reinforcement learning tasks. During surgery, subjects performed a two-alternative reinforcement learning task where they were asked to select between pairs of Japanese characters by pressing buttons on hand-held controllers. Immediately following each response, positive feedback (green screen, sound of cash register) or negative feedback (red screen, error tone) was probabilistically provided. An example positive and negative feedback trial are illustrated. Subjects were informed that each stimulus carried a distinct probability of reward and that their goal was to maximize positive feedback during the session. **(B)** Behavioral performance. During each session, subjects were presented with three stimulus pairs that varied in their relative reward rates (80/20, 70/30, and 60/40). To index subjects' learning during the task, we measured their bias toward selecting the high probability item during the final 10 trials of a given pair. Subjects reliably demonstrated a bias on the 80/20 pair (0.69) and a modest bias on the 70/30 pair (0.6). We did not observe a bias on the 60/40 pair (0.55). Error bars represent s.e.m across subjects. See main text for statistics.

Subjects performed a two-alternative probability learning task which has been previously used to study reinforcement learning and value-based decision making (Figure 2; Frank et al., 2004, 2007; Zaghloul et al., 2012). During the task, three pairs of symbols (denoted here by letters: AB, CD, EF) were presented in random order, and subjects were instructed to choose one of the two stimuli on each trial (Figure 2B). Selections were made by pressing buttons on handheld controllers placed in each hand. The three stimulus pairs were characterized by different relative rates of reward (AB, 80% vs. 20%; CD, 70% vs. 30%; EF, 60% vs. 40%). Reward rates associated with each symbol were determined randomly prior to each session and were fixed throughout the experiment. Probabilistic feedback followed each choice. In the event of positive feedback, the screen turned green, and the sound of a cash register was presented. In the event of negative feedback, the selection screen turned red, and an error tone was presented. Each trial consisted of presentation of the stimuli, subjects response, and a 2 s display of feedback. Subjects were asked to make selections which maximized their probability of obtaining positive feedback. As in previous reinforcement learning studies in the human SN (Zaghloul et al., 2009; Ramayya et al., 2014), there was no monetary payout and the provided feedback was virtual.

The rationale for including three item pairs with distinct relative reward rates is two-fold. First, we wanted to encourage learning throughout the session. Second, it allowed for the study of subthalamic nucleus neurons during decision conflict in a subsequent experiment. When possible, subjects first performed the task during the preoperative consultation, but in all cases, the task was reviewed with subjects on the morning of surgery. Further instructions were provided prior to beginning the task intra-operatively. During surgery, subjects performed the task on a laptop placed comfortably in front of them while the microelectrode was positioned in the SN. We aligned behavioral data with neural recordings by sending sync-pulses to the neural recording system from the behavioral laptop as participants performed the task. Some participants were bilaterally implanted with DBS electrodes and performed two intra-operative sessions of the task. The 25 subjects performed 32 sessions in total with a mean (±SD) of 123 (±7.1) trials per session. Each session typically lasted ≈ 15 minutes based on participants' response times.

### 2.3. Extracting neuronal spiking from microelectrode recordings

From each microelectrode recording, we extracted neuronal activity using the WaveClus software package (Quiroga et al., 2005). We band-pass filtered each voltage recording from 400 to 5000 Hz and manually removed periods of motion artifact. We identified spike events as positive or negative deflections in the voltage trace that crossed a threshold that was manually defined for each recording (≈4 SD about the mean amplitude of the filtered signal). The minimum duration between consecutive spike events (censor period) was set to be 1.5 ms. Spike events were subsequently clustered into units based on the first three principal components of the waveform. Noise clusters from motion artifact or power line contamination were manually invalidated. To ensure neuronal isolation, we filtered units based on established measures of isolation quality (IsoI; Neymotin et al., 2011). We rejected units if greater than 0.025 of their inter-spike intervals were refractory period violations (<3 ms) or if units were poorly separable from background noise in feature space (IsoI_*BG*_ < 4). If multiple units on a channel met the aforementioned criteria, but were poorly separated from each other (IsoI_*NN*_ < 4), they were considered together as a multi-unit, which is appropriate for our analyses because DA and GABA neurons are typically regionally clustered in the SN (Henny et al., 2012). We identified a total of 42 units. Seven units were excluded because of poor separation from background noise and/or refractory period violations. Of the remaining 35 units, two units were poorly separated from each other and were combined into a multi-unit. Thus, our dataset consisted of 33 single-units (IsoI_*BG*_ = 6.31 ± 1.13; mean ± SD), and one multi-unit (IsoI_*BG*_ = 7.53, mean IsoI_*NN*_ = 2.78). These data were identified from 17 of the 25 subjects; 18 sessions yielded one unit, whereas 8 sessions yielded two units.

### 2.4. Identifying putative DA and GABA activity

To understand the function of SN DA and GABA neurons, we sought to extract the activity of these neuronal populations from microelectrode recordings. Because *pars compacta* and *pars reticulata* are largely interspersed in the primate SN (Poirier et al., 1983), the location of the microelectrode relative to any anatomical landmarks is typically not used to isolate activity from these neuronal populations (also, see Menke et al., 2010). Instead, non-human primate electrophysiology studies usually identify putative DA and GABA units based on the properties of extracellular spike waveforms recorded on the microelectrode (Fiorillo et al., 2013). Previous studies which have combined electrophysiological recordings with pharmacological manipulations (Schultz and Romo, 1987) or histochemical techniques (Henny et al., 2012) have shown that DA neurons exhibit slow firing rates and broad waveforms, whereas GABA neurons display fast firing rates and narrow waveforms (Ungless and Grace, 2012). From each unit, we estimated baseline firing rate by computing the mean firing rate over the entire recording session and waveform duration by measuring the peak-to-trough duration (Barto et al., 2004). We identified putative DA units as those which displayed baseline firing rates slower than 15 Hz and waveform durations >0.8 ms, and GABA units as those which displayed baseline firing rates faster than 15 Hz and waveform durations <0.8 ms; similar parameters have been used in a prior non-human primate study (Matsumoto and Hikosaka, 2009). For the multi-unit in our dataset, we considered the baseline firing rate to be the average baseline firing rate of the two contributing units to account for the artificial elevation in firing rate that results from combining units.

For each DA and GABA unit, we computed smoothed firing rates during each trial by convolving the spike train with a Gaussian kernel (half-width = 75 ms). To aggregate firing rate responses across units, we computed normalized firing rate responses for each unit. Specifically, we computed a distribution of mean firing rates shown by the unit across all trials (0–1000 ms post-stimulus and −500–2000 ms surrounding response). We *z*-scored the smoothed firing rate during each trial based on the mean and standard deviation of this distribution. The time intervals used for the normalization process rarely overlapped because subjects demonstrated a mean reaction time of 2047 ms (±855 ms).

### 2.5. Statistical methods

For all statistical analyses, we aggregated activity within each unit and studied changes in firing rate across units. We studied firing rates from each unit in non-overlapping 250 ms windows (0–750 ms following stimulus presentation, and −500–1500 ms surrounding response trials), that were chosen *a priori* based on prior animal (Schultz et al., 1997; Cohen et al., 2012; Pan et al., 2013) and human (Zaghloul et al., 2009) studies of midbrain DA and GABA activity. To assess whether DA and GABA units demonstrated distinct temporal dynamics, we performed a 2 × 2 ANOVA following the three task events (stimulus presentation, responses resulting in positive and negative feedback). We considered time interval and neuron type to be fixed effects. To account for variability that may result from obtaining multiple samples from each population, we included neuron number as random effect nested within the neuron-type fixed effect. We performed *post-hoc t*-tests to identify specific changes in neural activity, and corrected for multiple comparisons using a false-discovery rate (FDR) procedure.

## 3. Results

We obtained microelectrode recordings from the substantia nigra (SN) of 25 patients (16 males, mean age = 57.36) undergoing deep brain stimulation surgery for the treatment of Parkinson's disease (PD). As per routine clinical procedure, microelectrodes were advanced into the substantia nigra (SN) in order to identify the inferior border of the subthalamic nucleus, the target for the stimulating electrode (Figure 1A; Jaggi et al., 2004; Zaghloul et al., 2009). From each SN recording, we extracted neuronal spiking activity and identified putative DA (*n* = 13, mean rate = 4.56 Hz, mean duration = 0.87 ms) and GABA (*n* = 10, mean rate = 25.0 Hz, mean duration = 0.62 ms) units based on their baseline firing rates and waveform durations (Materials and Methods, Figure 1B).

As we obtained recordings, subjects performed a two-alternative probability learning task where they were asked to select between pairs of Japanese characters by pressing buttons on hand-held controllers. Immediately following each response, they probabilistically received positive or negative feedback (Figure 2A). Each stimulus carried a distinct probability of reward and each pair always consisted of a high-probability and a low-probability stimulus. During each session, subjects were presented with three item pairs that varied in their relative reward rates (80/20, 70/30, and 60/40). Subjects were instructed to select stimuli that maximized their probability of receiving positive feedback. To index learning on a particular item pair, we measured the tendency that subjects demonstrated toward selecting the high-probability item during the last 10 presentations of that item pair (Figure 2B). We found that subjects reliably demonstrated such a tendency during the 80/20 pair [0.69, *t*_(30)_ = 4.64, *p* < 0.001]. Subjects showed a trend toward such a tendency on the 70/30 pair (0.60, *p* = 0.08), but not on the 60/40 item pair (0.55, *p* > 0.2).

**Figure 3:**
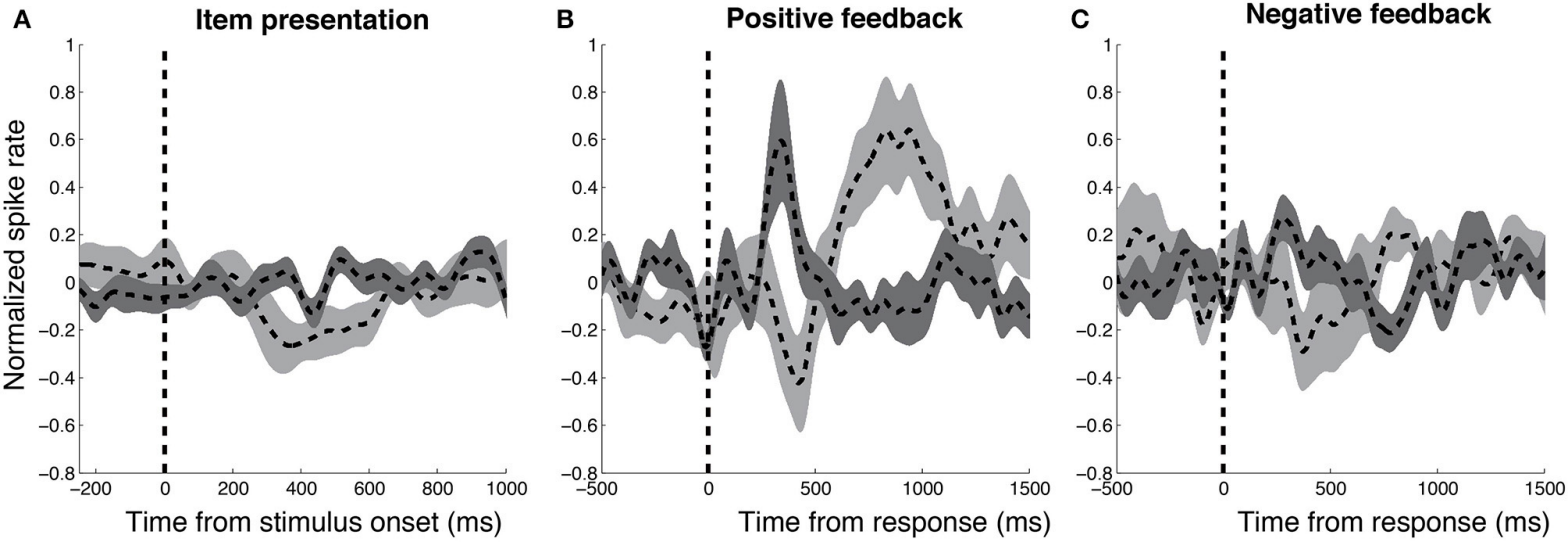
**Distinct responses from DA and GABA units following positive feedback**. We studied aggregate normalized firing rates from DA (*n* = 13, dark gray) and GABA (*n* = 10, light gray) units in relation to three task events—stimulus presentation (**A**) subject responses that resulted in positive feedback **(B)**, and negative feedback **(C)**. We observed distinct responses from DA and GABA units following responses associated with positive feedback, but not following stimulus presentation or responses associated with negative feedback. Firing rate responses were smoothed using a Gaussian-kernel (half-width = 75 ms). Width of each response represents s.e.m across units.

To compare the functional properties of DA and GABA units, we studied aggregate normalized firing rates from each population aligned to three task-related events—stimulus presentation, responses associated with positive feedback, and responses associated with negative feedback (Figure 3). We separately examined neural activity following responses associated with positive and negative feedback because DA units have been shown to demonstrate opposing responses during these trials (Zaghloul et al., 2009). To compare responses from the two groups during these three conditions, we binned firing rates from each unit in non-overlapping 250 ms windows (0–750 ms following stimulus presentation, and −500–1500 ms surrounding response trials) and applied two-factor ANOVAs with neuron-type and time-interval as fixed effects. We included neuron number as random effect nested within the neuron-type fixed effect to account for variability that occurs when obtaining multiple samples from each population (see Statistical Methods). Following positive feedback presentation, we observed a significant interaction between neuron-type and time-interval [*F*_(7, 183)_ = 6.02, Mean Squared Error (MSE) = 0.81, *p* < 0.001] suggesting that DA and GABA neurons demonstrated distinct temporal dynamics during these trials. *Post-hoc t*-tests revealed that DA units demonstrated greater firing rates than GABA units during the 250–500 ms time interval [*t*_(21)_ = 2.37, *p* = 0.028] whereas GABA units demonstrated greater firing rates than DA units during the 500–750 and 750–1000 ms time intervals [*t*_(21)_'s > 2.52, *p*'s < 0.029; false-discovery rate (FDR) corrected *p*'s < 0.07]. We did not observe significant interactions between neuron-type and time-interval during stimulus presentation or following negative feedback (*p*'s > 0.16). Thus, we observed distinct responses from DA and GABA neurons following positive feedback presentation, but not following stimulus or negative feedback presentation.

**Figure 4:**
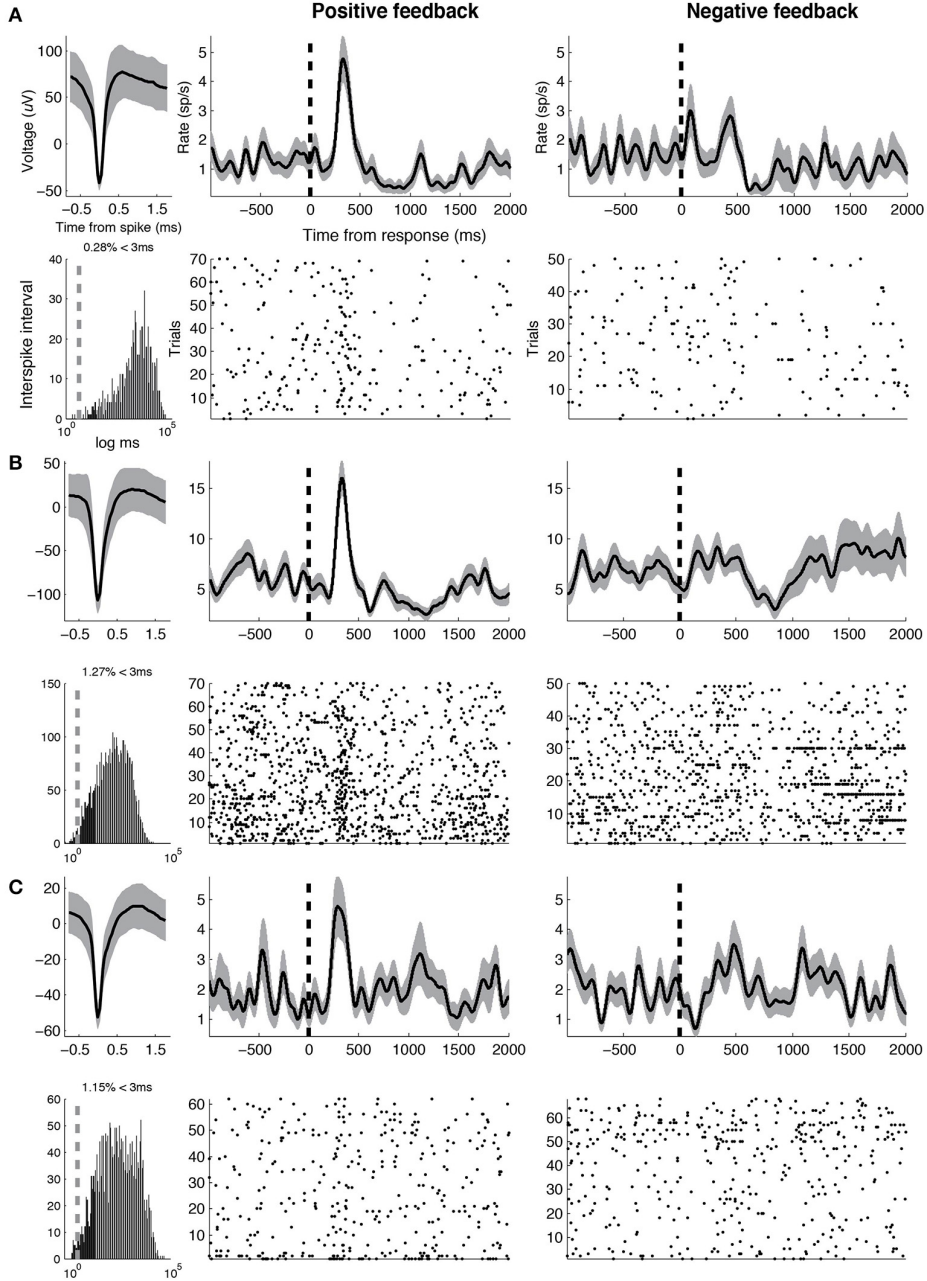
**Example DA units**. Three representative DA units are shown. For each unit, average waveform (top left), inter-spike intervals on a logarithmic time scale (bottom left, vertical line indicates 3 ms), smoothed rate (half-width = 75 ms) and raster following responses associated with positive (middle) and negative feedback (right), respectively, (vertical line indicates response). Width of smooth rate represents s.e.m. Baseline firing rates, waveform durations for the three units are as follows. **(A)** 1.31 Hz, 0.82 ms **(B)** 6.91 Hz, 0.85 ms **(C)** 3.35 Hz, 0.92 ms.

**Figure 5:**
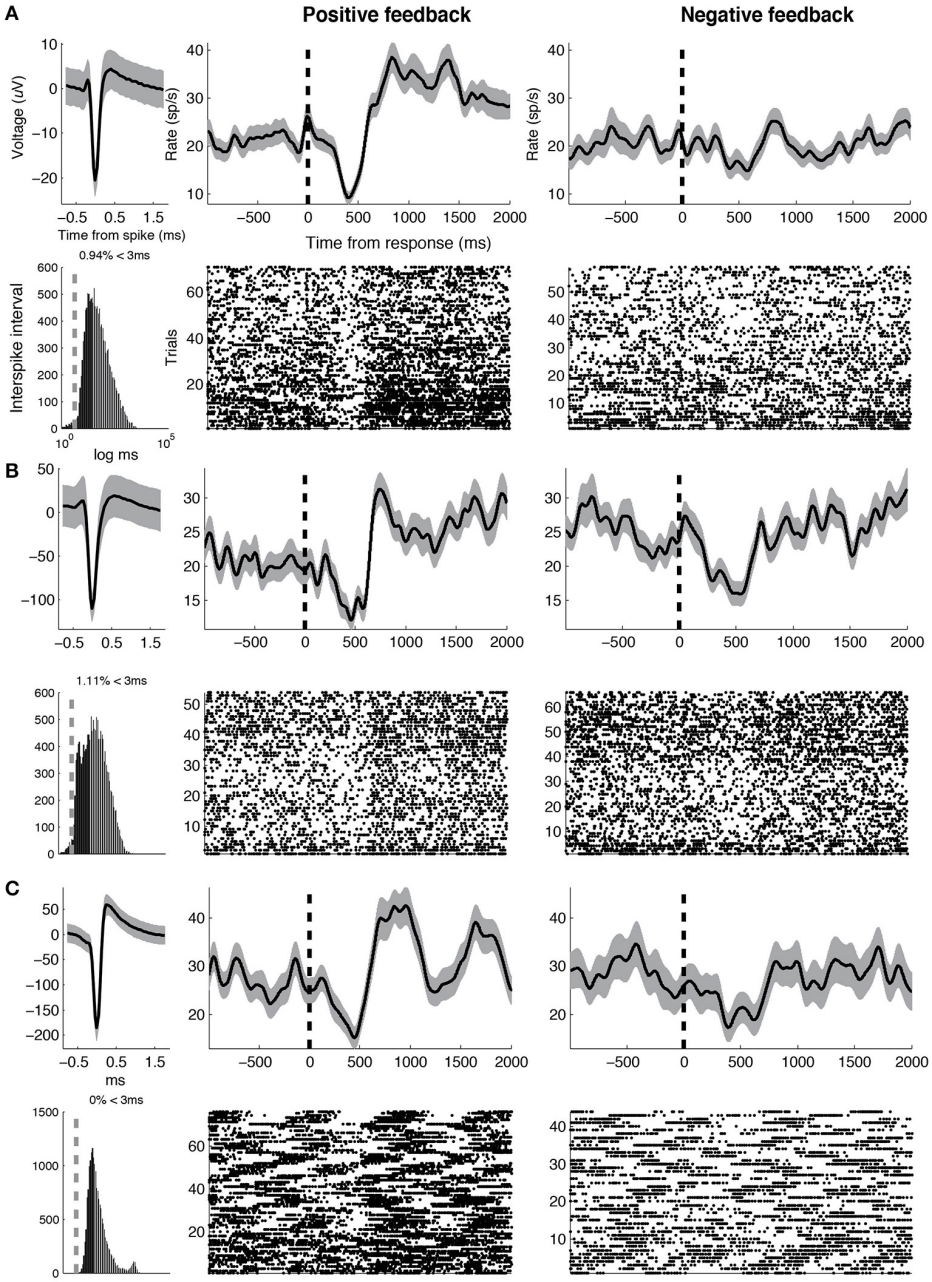
**Example GABA units**. Three representative GABA units are shown. Same conventions as in Figure 4. Baseline firing rates and waveform durations are as follows. **(A)** 25.6 Hz, 0.67 ms **(B)** 27.0 Hz, 0.75 ms **(C)** 28.3 Hz, 0.39 ms.

To assess whether differences between DA and GABA firing rates following positive feedback were driven by changes in DA activity, GABA activity or both, we studied changes in each population's firing rates from baseline. We selected the following time intervals of interest based on the results of the previous analysis: 250–500 ms (“early,” when DA activity was greater than GABA activity) and 500-1000 ms (“late,” when GABA activity was greater than DA activity). For DA units, we observed increased firing rate from baseline during the early time interval [*t*_(12)_ = 2.15, *p* = 0.052], but did not observe significant changes in firing during the late time interval (*p* > 0.2). For GABA units, we observed the opposite pattern—we did not observe significant changes in firing during the early time interval (*p* > 0.2), but observed significant increases in firing rate during the late time interval [*t*_(9)_ = 3.29, *p* = 0.009]. Thus, the major changes in neural activity following positive feedback presentation included an early increase in DA activity and a late increase in GABA activity. Example DA and GABA units are shown in Figures 4, 5, respectively.

## 4. Discussion

We studied neuronal activity in the SN of patients undergoing DBS surgery for the treatment of Parkinson's disease as they performed a two-alternative reinforcement learning task. During each trial of the task, subjects were presented with a pair of stimuli, selected one of the stimuli by pressing buttons on hand-held controllers (“response”), and immediately received positive or negative audio-visual feedback. We identified putative DA and GABA neurons based on the physiological properties of their extracellular waveforms, and compared the functional properties of the two populations during the task.

### 4.1. DA and GABA neurons in the human SN are functionally distinct

Our main finding was that DA and GABA neurons demonstrated distinct temporal dynamics following responses that resulted in positive feedback. Whereas DA neurons demonstrated phasic bursts in activity (250–500 ms post-feedback), GABA neurons demonstrated more delayed and sustained increases in activity (500–1000 ms post-feedback). These results provide the first electrophysiological evidence for a functional dissociation between DA and GABA neurons in the human SN. Whereas prior histochemical studies have shown that DA and GABA neurons co-exist in the human SN (Damier et al., 1999a), the only direct evidence for a functional dissociation between these neural populations has come from animal electrophysiology studies (DeLong et al., 1983; Schultz et al., 1997). Our findings provide a bridge between these studies by demonstrating a functional dissociation between these neural populations in the human SN. As such, our results provide electrophysiological support for neuro-computational theories of human basal ganglia function that ascribe distinct roles to these neural populations during learning and decision-making (Bogacz and Gurney, 2007).

### 4.2. Functional significance of phasic DA bursts

Animal electrophysiology studies have shown that DA neurons demonstrate phasic bursts of activity that correlate with reward prediction errors (Schultz et al., 1997; Bayer and Glimcher, 2005). Enhancement of these DA bursts via electrical microstimulation (Reynolds et al., 2001) or optogenetics (Tsai et al., 2009) results in enhanced learning, suggesting a causal relation between phasic DA bursts and learning. However, several factors limit the generalizability of these studies to human behavior. First, animal learning is typically studied following primary rewards and punishments (e.g., juice and airpuffs), whereas human learning is often motivated by higher-order abstract rewards (e.g., rational and social goals). Second, animals in these studies have typically undergone long periods of intense training, whereas much of human learning occurs in novel situations.

Recent studies in patients undergoing DBS surgery for Parkinson's disease suggest a functional role for phasic DA bursts in human reinforcement learning. Zaghloul et al. (2009) demonstrated reward prediction error-like responses in a subset of SN neurons that electrophysiologically resemble DA neurons described in animal studies (putative DA neurons). The current study functionally validates the use of these electrophysiological criteria by showing that putative DA neurons demonstrate distinct post-reward responses from other neurons in the region. Consistent with our findings, Ramayya et al. (2014) found that microstimulation applied near SN neuronal populations that showed post-reward bursts of activity and broad waveforms resulted in altered learning. Generally, our finding that putative DA neurons demonstrated post-reward bursts in activity (Figure 4) is consistent with their hypothesized role in providing reinforcement following rewards (Glimcher, 2011).

Similar to the Zaghloul et al. (2009) study, we observed DA bursts 250–500 ms following feedback, which is later than DA bursts typically observed in animal studies (100–250 ms; Niv and Montague, 2008). The more delayed latency might be attributed to the presentation of abstract audio-visual rewards, rather than primary rewards, each of which might engage DA neurons through distinct processes (prefrontal vs. brainstem mechanisms, respectively, Glimcher, 2011). Unlike the Zaghloul et al. (2009) study, however, we did not observe clear evidence that post-reward DA bursts represented a reward prediction error (although, see Supplementary Material). This may be because subjects demonstrated limited learning during the task. Additionally, whereas Zaghloul et al. (2009) observed DA pauses during the 150–300 ms post-feedback interval, we did not observe reliable decreases in activity across DA neurons (although, see Figure 4B). This discrepancy may be explained by the fact that the negative feedback condition in the Zaghloul et al. (2009) study was associated with an absence of reward, whereas in our study, it was associated with the presentation of a salient negative stimulus. Previous animal studies have shown that pauses in DA activity are less frequently observed following the presentation of aversive, salient stimuli (Matsumoto and Hikosaka, 2009).

### 4.3. Functional significance of GABA activity

In contrast to DA neurons, GABA neurons demonstrated delayed, and sustained increases in activity following positive feedback. These patterns are consistent with findings from animal studies that have shown sustained changes in midbrain GABA activity following visual stimulus and reward presentation (Handel and Glimcher, 2000; Sato and Hikosaka, 2002; Joshua et al., 2009; Cohen et al., 2012). We speculate that these post-feedback GABA responses are related to a reciprocal interaction with DA neurons. Previous work has shown that GABA neurons demonstrate increased firing rates when exposed to dopamine (Waszczak and Walters, 1983), suggesting that DA neurons may exert excitatory control of GABA firing. Conversely, SN GABA neurons exhibit inhibitory projections onto midbrain DA neurons, and may exert inhibitory control over DA neurons (Tepper et al., 1995; Lobb et al., 2011; Henny et al., 2012; Pan et al., 2013). Then, following a phasic DA burst, GABA neurons might display an increase in firing rate that might act to regulate DA firing and suppress subsequent DA phasic bursts. GABA responses might be more prominent following positive compared to negative feedback if the majority of DA neurons that provide inputs to GABA neurons demonstrate preferential increases in phasic activity following positive feedback compared to negative feedback. Although the majority of SN GABA neurons reside in the *pars reticulata*, a subset of GABA neurons are also known to exist in the *pars compacta* region (Nair-Roberts et al., 2008; Ungless and Grace, 2012).

Some GABA neurons also demonstrated robust pauses in activity soon after feedback was presented (see Figure 5). Pauses in GABA-ergic activity typically suggest a release of inhibition on downstream structures, and have been classically observed during movement and saccade generation (DeLong et al., 1983; Hikosaka and Wurtz, 1983). These pauses in activity are thought to decrease inhibition on (“disinhibit”) downstream motor structures (e.g., superior colliculus; Carpenter et al., 1976), and allow for the execution of a movement. Thus, the observed GABA pauses may be related to some movement expressed by subjects immediately following the presentation of salient sensory stimuli during the feedback condition (possibly orienting saccades; Hikosaka and Wurtz, 1983). However, we are unable to test this hypothesis because we did not monitor eye movements during the study. Alternatively, the observed pauses in GABA activity may be related to decreased inhibition on DA neurons that would facilitate post-feedback DA bursting (Luscher and Ungless, 2006; Lobb et al., 2011).

### 4.4. Limitations

We note several limitations to our study. First, we are unable to provide direct histochemical evidence that these electrophysiologically-identified neural subgroups reflect distinct neuronal populations. However, there is a large body of evidence from animal studies suggesting that these electrophysiological criteria may be used to identify distinct midbrain neuronal populations (Ungless and Grace, 2012). As such, several animal studies rely on electrophysiological criteria alone to identify functional subpopulations within the midbrain (Matsumoto and Hikosaka, 2009; Fiorillo et al., 2013). Second, the population we studied in this experiment–patients undergoing DBS for Parkinson's disease–is known to have degeneration of neurons in SN. Ideally, one would like to study the function of SN neurons in healthy human subjects, but at present such recordings may not be ethically obtained in any other human population. Converging evidence from histochemical (Damier et al., 1999b) and electrophysiological studies (Zaghloul et al., 2009; Ramayya et al., 2014) in patients with Parkinson's disease and in animals (Hollerman and Grace, 1990; Zigmond et al., 1990; Wang et al., 2010) indicate that a significant population of viable DA neurons remain in the Parkinsonian SN. We suggest that the observed DA and GABA responses reflect activity from the subpopulation of healthy neurons that remain in the SN.

## Author contributions

Kareem A. Zaghloul, Christoph T. Weidemann, Gordon H. Baltuch, and Michael J. Kahana designed research; Ashwin G. Ramayya, Kareem A. Zaghloul, Christoph T. Weidemann, and Gordon H. Baltuch performed research; Ashwin G. Ramayya analyzed the data; Ashwin G. Ramayya and Michael J. Kahana wrote the paper. All authors contributed to the intellectual content of the research and provided final approval on the manuscript.

### Conflict of interest statement

The authors declare that the research was conducted in the absence of any commercial or financial relationships that could be construed as a potential conflict of interest.

## References

[B1] Barto A. Singh S. Chentanez N. (2004). Intrinsically motivated learning of hierarchical collections of skills, in Proceedings of the 3rd International Conference on Development and Learning (San Diego, CA: Salk Institute).

[B2] Bayer H. Glimcher P. (2005). Midbrain dopamine neurons encode a quantitative reward prediction error signal. Neuron 47, 129–141 10.1016/j.neuron.2005.05.020 15996553 PMC1564381

[B3] Bogacz R. Gurney K. (2007). The basal ganglia and cortex implement optimal decision making between alternative actions. Neural Comput. 19, 442–477 10.1162/neco.2007.19.2.442 17206871

[B4] Carpenter M. B. Nakano K. Kim R. (1976). Nigrothalamic projections in the monkey demonstrated by autoradiographic technics. J. Comp. Neurol. 165, 401–415 10.1002/cne.901650402 57125

[B5] Cohen J. Haesler S. Vong L. Lowell B. Uchida N. (2012). Neuron-type-specific signals for reward and punishment in the Ventral Tegmental Area. Nature 482, 85–88 10.1038/nature10754 22258508 PMC3271183

[B6] Damier P. Hirsch E. Agid Y. Graybiel A. M. (1999a). The substantia nigra of the human brain i. nigrosomes and the nigral matrix, a compartmental organization based on calbindin d28k immunohistochemistry. Brain 122, 1421–1436 10.1093/brain/122.8.1421 10430829

[B7] Damier P. Hirsch E. Agid Y. Graybiel A. M. (1999b). The substantia nigra of the human brain ii. patterns of loss of dopamine-containing neurons in Parkinson's disease. Brain 122, 1437–1448 10.1093/brain/122.8.1437 10430830

[B8] DeLong M. Crutcher M. Georgopoulos A. P. (1983). Relations between movement and single cell discharge in the substantia nigra of the behaving monkey. J. Neurosci. 3, 1599–1606 6875659 10.1523/JNEUROSCI.03-08-01599.1983 PMC6564529

[B9] Fiorillo C. Yun S. Song M. (2013). Diversity and homogeneity in responses of midbrain dopamine neurons. J. Neurosci. 33, 4693–4709 10.1523/JNEUROSCI.3886-12.2013 23486943 PMC3873403

[B10] Frank L. M. Stanley G. Brown E. (2004). Hippocampal plasticity across multiple days of exposure to novel environments. J. Neurosci. 24, 7681–7689 10.1523/JNEUROSCI.1958-04.2004 15342735 PMC6729632

[B11] Frank M. Samanta J. Moustafa A. Sherman S. (2007). Hold your horses: impulsivity, deep brain stimulation, and medication in parkinsonism. Science 318, 1309–1312 10.1126/science.1146157 17962524

[B12] Glimcher P. (2011). Understanding dopamine and reinforcement learning: the dopamine reward prediction error hypothesis. Proc. Natl. Acad. Sci. U.S.A. 108, 15647–15654 10.1073/pnas.1014269108 21389268 PMC3176615

[B13] Handel A. Glimcher P. W. (2000). Contextual modulation of substantia nigra pars reticulata neurons. J. Neurophysiol. 83, 3042–3048 10805699 10.1152/jn.2000.83.5.3042

[B14] Henny P. Brown M. Northrop A. Faunes M. Ungless M. Magill P. (2012). Structural correlates of heterogeneous *in vivo* activity of midbrain dopaminergic neurons. Nat. Neurosci. 15, 613–619 10.1038/nn.3048 22327472 PMC4242968

[B15] Hikosaka O. Wurtz R. (1983). Visual and oculomotor functions of monkey substantia nigra pars reticulata. i. relation of visual and auditory responses to saccades. J. Neurophysiol. 49, 1230–1253 6864248 10.1152/jn.1983.49.5.1230

[B16] Hollerman J. Grace A. (1990). The effects of dopamine-depleting brain lesions on the electrophysiological activity of rat Substantia Nigra dopamine neurons. Brain Res. 533, 203–212 10.1016/0006-8993(90)91341-D 2126975

[B17] Jaggi J. Umemura A. Hurtig H. Siderowf A. Colcher A. Stern M. (2004). Bilateral subthalamic stimulation of the subthalamic nucleus in Parkinson's disease: surgical efficacy and prediction of outcome. Stereotact. Funct. Neurosurg. 82, 104–114 10.1159/000078145 15305083

[B18] Joshua M. Adler A. Rosin B. Vaadia E. Bergman H. (2009). Encoding of probabilistic rewarding and aversive events by pallidal and nigral neurons. J. Neurophysiol. 101, 758–772 10.1152/jn.90764.2008 19052110

[B19] Lobb C. Wilson C. Paladini C. (2011). High-frequency, short-latency disinhibition bursting of midbrain dopaminergic neurons. J. Neurophysiol. 105, 2501–2511 10.1152/jn.01076.2010 21367999 PMC3094190

[B20] Luscher C. Ungless M. (2006). The mechanistic classification of addictive drugs. PLoS Med. 3:e437 10.1371/journal.pmed.0030437 17105338 PMC1635740

[B21] Matsumoto M. Hikosaka O. (2009). Two types of dopamine neuron distinctly convey positive and negative motivational signals. Nature 459, 837–841 10.1038/nature08028 19448610 PMC2739096

[B22] Menke R. Jbabdi S. Miller K. Matthews P. Zarei M. (2010). Connectivity-based segmentation of the Substantia Nigra in human and its implications in Parkinson's disease. Neuroimage 52, 1175–1180 10.1016/j.neuroimage.2010.05.086 20677376

[B23] Moyer J. T. Danish S. F. Keating J. G. Finkel L. H. Baltuch G. H. Jaggi J. L. (2007). Implementation of dual simultaneous microelectrode recording systems during deep brain stimulation surgery for Parkinson's disease: technical note. Neurosurgery 60, E177–E178 10.1227/01.NEU.0000249250.40676.7E 17297356

[B24] Nair-Roberts R. Chatelain-Badie S. Benson E. White-Cooper H. Bolam J. Ungless M. (2008). Stereological estimates of dopaminergic, GABA-ergic, and glutamatergic neurons in the Ventral Tegmental Area, Substantia Nigra and Retrorubal Field in the rat. J. Neurosci. 152, 1024–1031 10.1016/j.neuroscience.2008.01.046 18355970 PMC2575227

[B25] Neymotin S. Lyton W. Olypher A. V. Fenton A. A. (2011). Measuring the quality of neuronal identification in ensemble recordings. J. Neurosci. 31, 16398–16409 10.1523/JNEUROSCI.4053-11.2011 22072690 PMC3247202

[B26] Niv Y. Montague P. R. (2008). Theoretical and empirical studies of learning, in Neuroeconomics: Decision Making and the Brain, Chapter 22, eds Glimcher P. W. Camerer C. F. Fehr E. Poldrack R. A. (London: Academic Press), 329–350

[B27] Pan W. X. Brown J. Dudman J. (2013). Neural signals of extinction in the inhibitory microcircuit of the ventral midbrain. Nat. Neurosci. 16, 71–78 10.1038/nn.3283 23222913 PMC3563090

[B28] Poirier L. Giguére M. Marchand R. (1983). Comparative morphology of the substantia nigra and ventral tegmental area in the monkey, cat and rat. Brain Res. Bull. 11, 371–397 10.1016/0361-9230(83)90173-9 6640366

[B29] Quiroga R. Q. Reddy L. Kreiman G. Koch C. Fried I. (2005). Invariant visual representation by single neurons in the human brain. Nature 435, 1102–1107 10.1038/nature03687 15973409

[B30] Ramayya A. G. Misra A. Baltuch G. H. Kahana M. J. (2014). Microstimulation of the human substantia nigra following feedback alters reinforcement learning. J. Neurosci. 34, 6887–6895 10.1523/JNEUROSCI.5445-13.2014 24828643 PMC4019802

[B31] Rescorla R. Wagner A. (1972). A theory of pavolvian conditioning: variations in the effectiveness of reinforcement and nonreinforcement, in Classical Conditioning II: Current Research and Theory, eds Black A. Prokasy W. (New York, NY: Appleton Century Crofts), 64–99

[B32] Reynolds J. Hyland B. Wickens J. (2001). A cellular mechanism of reward-related learning. Nature 413, 67–70 10.1038/35092560 11544526

[B33] Sato M. Hikosaka O. (2002). Role of primate substantia nigra pars reticulata in reward-oriented saccadic eye movement. J. Neurosci. 22, 2363–2373 11896175 10.1523/JNEUROSCI.22-06-02363.2002 PMC6758246

[B34] Schultz W. Dayan P. Montague P. R. (1997). A neural substrate of prediction and reward. Science 275, 1593–1599 10.1126/science.275.5306.1593 9054347

[B35] Schultz W. Romo R. (1987). Responses of nigrostriatal dopamine neurons to high-intensity somatosensory stimulation in the anesthetized monkey. J. Neurophysiol. 57, 201–217 3559672 10.1152/jn.1987.57.1.201

[B36] Tepper J. Martin L. Anderson D. (1995). GABA-A receptor-mediated inhibition of rat Substantia Nigra dopaminergic neurons by pars reticulata projection neurons. J. Neurosci. 15, 3092–3103 7722648 10.1523/JNEUROSCI.15-04-03092.1995 PMC6577766

[B37] Tsai H. Zhang F. Adamatidis A. Stuber G. Bonci A. Lecea L. (2009). Phasic firing in dopaminergic neurons is sufficient for behavioral conditioning. Science 324, 1080–1084 10.1126/science.1168878 19389999 PMC5262197

[B38] Ungless M. Grace A. (2012). Are you or aren't you? Challenges associated with physiologically identifying dopamine neurons. Trends Neurosci. 35, 422–430 10.1016/j.tins.2012.02.003 22459161 PMC3383926

[B39] Wang Y. Zhang Q. Ali U. Gui Z. Hui Y. Chen L. (2010). Changes in firing rate and pattern of GABA-ergic neurons in subregions of the Substantia Nigra pars reticulata in rat models of Parkinson's Disease. Brain Res. 1324, 54–63 10.1016/j.brainres.2010.02.008 20149784

[B40] Waszczak B. Walters J. (1983). Dopamine modulation of the effects of gamma-aminobutyric acid on Substantia Nigra pars reticulata neurons. Science 220, 218–221 10.1126/science.6828891 6828891

[B41] Zaghloul K. A. Blanco J. A. Weidemann C. T. McGill K. Jaggi J. L. Baltuch G. H. (2009). Human Substantia Nigra neurons encode unexpected financial rewards. Science 323, 1496–1499 10.1126/science.1167342 19286561 PMC2839450

[B42] Zaghloul K. A. Lega B. C. Weidemann C. T. Jaggi J. L. Baltuch G. H. Kahana M. J. (2012). Neuronal activity in the human Subthalamic Nucleus encodes decision conflict during action selection. J. Neurosci. 32, 2453–2460 10.1523/JNEUROSCI.5815-11.2012 22396419 PMC3296967

[B43] Zigmond M. Abercrombie E. Berger T. W. Grace A. Stricker E. (1990). Compensations after lesions of central dopaminergic neurons: some clinical and basic implications. Trends Neurosci. 13, 290–296 10.1016/0166-2236(90)90112-N 1695406

