# Supplementary material for: Electrophysiological evidence for functionally distinct neuronal populations in the human substantia nigra
Source: Front Hum Neurosci. 2014 Sep 9;8:655. doi: 10.3389/fnhum.2014.00655 (PMC4158808; doi:10.3389/fnhum.2014.00655)
Supplement: Supplementary file 1 [file Presentation1.PDF]

Frontiers in Human Neuroscience

Research Article
15 August 2014

Supplemental Data: Electrophysiological evidence for functionally distinct neuronal populations in the human substantia nigra

Ashwin G. Ramayya [1], Kareem A. Zaghloul [3], Christoph T. Weidemann,[4],Gordon H. Baltuch,[5,*], and Michael J. Kahana,[2*]

[1] *Neuroscience Graduate Group,* [2] *Dept. of Psychology, University of Pennsylvania, Philadelphia, PA 19104*
[3] *Surgical Neurology Branch, NINDS, National Institutes of Health, Bethesda, Maryland 20892*
[4] *Department of Psychology, Swansea University, Swansea, United Kingdom, SA2 8PP*
[5] *Department of Neurosurgery, Perelman School of Medicine, University of Pennsylvania, Philadelphia, PA 19104*
[*] *These authors contributed equally to this work*

Correspondence*:
Gordon H. Baltuch
Department of Neurosurgery, Perelman School of Medicine, 235 South 8th Street, Philadelphia, PA 19106, baltuchg@mail.med.upenn.edu

Michael J. Kahana
Department of Psychology, University of Pennsylvania, 3401 Walnut St., Room 303C, Philadelphia, PA 19104, kahana@psych.upenn.edu

Comparing DA and GABA responses following positive and negative feedback To shed light on the functional properties of DA and GABA neurons, we compared their firing rates following positive and negative feedback obtained during the early and late time intervals, respectively. For DA neurons, we did not observe significant differences in activity following the two feedback conditions ($p > 0.14$). Thus, although individual DA neurons demonstrated differential activity following positive and negative feedback (**Figure 4**), we did not observe reliable differences across the population of DA neurons, which may be due to a lack of power. For GABA neurons, we observed a trend towards greater firing rates following positive compared to negative feedback during the late time interval ($t(9) = 2.24, p = 0.052$). If GABA responses reflect a reciprocal interaction with DA neurons (see *Discussion*), more prominent tonic GABA responses following positive feedback might suggest that excitatory DA inputs onto these neurons are stronger following positive compared to negative feedback.

Relating post-reward DA bursts to reward prediction error Theories of learning posit that decisions are altered based on a reward prediction error, or the mismatch between obtained and expected rewards (**Rescorla and Wagner**, 1972). Previous studies have shown that DA neurons encode a reward prediction error because they selectively show post-reward bursts in activity when rewards are unexpected (**Schultz et al.**, 1997; **Zaghloul et al.**, 2009). Because subjects demonstrated poor learning during the task (**Figure 2**), the vast majority of rewards obtained during the task were unlikely to be predicted based on past experience, and would be classified as “unexpected.” Thus, we were limited in our ability to evaluate whether post-reward DA bursts represented a reward prediction error.

Our behavioral analyses suggested that subjects demonstrated evidence of learning on the 80/20 pair, but not the 70/30, or the 60/40 pair (see *Results*, **Figure 2**). Thus, rewards obtained during the last 10 trials of the 80/20 pair would be better predicted by subjects than those obtained during the first 10 trials. To assess whether post-reward DA bursts reflected a reward prediction error, we compared DA activity during the 250-500 ms post-feedback interval following rewards obtained during the first 10 trials of the 80/20 pair ("unexpected"), and those obtained during the last 10 trials of the 80/20 pair ("expected"). We observed greater phasic DA activity during the unexpected reward condition compared to the expected condition ($t(22) = 2.49, p = 0.02$), which is consistent with a reward prediction error. We did not observe significant differences between phasic DA activity obtained during early and late reward trials associated with the other item pairs (p's> 0.4), or following negative feedback or stimulus presentation (p's> 0.15). Also, we did not observe reliable differences in tonic GABA activity (500-1000 ms) during early and late trials, following positive feedback, negative feedback, or stimulus presentation associated with the 80/20 condition (p's> 0.29).

REFERENCES

Rescorla, R. and Wagner, A. (1972), A theory of pavolvian conditioning: Variations in the effectiveness of reinforcement and nonreinforcement, in A. Black and W. Prokasy, eds., Classical Conditioning II: Current Research and Theory (Appleton Century Crofts, New York), 64–99

Schultz, W., Dayan, P., and Montague, P. R. (1997), A neural substrate of prediction and reward, *Science*, 275, 1593–1599

Zaghloul, K. A., Blanco, J. A., Weidemann, C. T., McGill, K., Jaggi, J. L., Baltuch, G. H., et al. (2009), Human Substantia Nigra neurons encode unexpected financial rewards, *Science*, 323, 1496–1499
